# Editorial: Puberty: neurologic and physiologic development

**DOI:** 10.3389/fendo.2023.1258656

**Published:** 2023-07-25

**Authors:** Duarte Henriques-Neto, Miguel Peralta, Adilson Marques

**Affiliations:** ^1^ Research Centre in Sports Sciences, Health Sciences and Human Development, University Institute of Maia, Maia, Portugal; ^2^ Interdisciplinary Center for Human Performance, Faculty of Human Kinetics, University of Lisbon, Dafundo, Portugal

**Keywords:** puberty, physiology development, neurologic, endocrine, brain

## 1 Introduction

Puberty is characterised as the endpoint of childhood and the beginning of adolescence. All physiological and neurological changes represent a pivotal phase in human development, transitioning from childhood to adulthood. During this stage, there is a profound and significant biological interaction among various human systems as they mature. The harmonious functioning of different biological systems through hormonal, physical, and neurological processes is crucial for this phase of human development. These biological systems’ functioning depends on an individual’s genetic heritage and their social life as an adolescent (e.g., family support, socioeconomic status, and healthy behaviours) (1–4).

In girls, puberty onset begins around 11 years old, while in boys it occurs at around 12 years old. During this time, the first anatomical transformations occur, such as the development of breast buds in girls and an increase in testicular volume in boys (4). The hypothalamic-pituitary-gonadal axis undergoes significant activation and maturation during puberty, leading to the secretion of sex hormones, including testosterone and estrogen. These hormonal changes influence the development of secondary sexual characteristics, reproductive organs, and overall body growth and maturation (5). During puberty, the hypothalamus, a region of the brain, begins to release gonadotropin-releasing hormone (GnRH), which stimulates the pituitary gland to release two important hormones: luteinising hormone (LH) and follicle-stimulating hormone (FSH) (6). These hormones act on the ovaries in females or the testes in males, triggering the production of sex hormones—estrogen in females and testosterone in males (6).

The puberty period depends on genetics and social factors, such as nutrition, socioeconomic status, and psychological characteristics (4, 5). This period, driven by hormonal fluctuations and genetic factors, contributes to the cognitive and behavioural transformations observed during adolescence, typically occurring 2-4 years after gonadarche (4, 7). Structural and functional reorganisation in the brain affects areas responsible for emotion regulation, social cognition, and decision making. Earlier pubertal timing has been associated with accelerated brain development, particularly in subcortical and frontal regions in females and subcortical regions in males (7).

## 2 Testosterone enanthate and testosterone undecanoate treatment

### 2.1 Medical applications

Testosterone enanthate and testosterone undecanoate are exogenous forms of testosterone used for various therapeutic purposes, including testosterone replacement therapy and managing delayed puberty. These treatments can help normalise hormone levels and facilitate appropriate pubertal development in individuals with hormonal imbalances.

### 2.2 Considerations and outcomes

Careful monitoring and individualised treatment plans are essential to optimise the benefits and minimise potential side effects of testosterone enanthate and undecanoate treatment. These therapies can positively impact pubertal development in individuals with testosterone deficiency, leading to improvements in physical and psychosocial well-being (Osterbrand et al.).

## 3 Untargeted metabolomics and lipidomics in girls with central precocious puberty

### 3.1 Exploring metabolic alterations

Untargeted metabolomics and lipidomics techniques provide valuable insights into the metabolic changes associated with central precocious puberty in girls. These approaches enable identifying and characterising specific metabolites and lipid profiles that may contribute to the pathophysiology of early pubertal onset.

### 3.2 Potential biomarkers and mechanisms

Comprehensive metabolomic and lipidomic analyses may aid in the identification of biomarkers for early detection and monitoring of central precocious puberty. Moreover, these studies can shed light on the underlying molecular mechanisms involved in the neuroendocrine regulation of pubertal onset (Zhao et al.).

## 4 Precocious puberty under stressful conditions after the COVID-19 epidemic

### 4.1 Psychosocial impact

The COVID-19 pandemic has imposed unprecedented stressors on individuals and communities worldwide. Emerging evidence suggests a potential link between stressful conditions during and after the pandemic and an increased prevalence of precocious puberty. The psychological and physiological consequences of stress may disrupt normal pubertal timing and lead to the early onset of puberty.

### 4.2 Implications and future directions

Understanding the influence of stressful events, such as the COVID-19 epidemic, on pubertal development is crucial for developing appropriate support systems and interventions to mitigate the potential negative effects. Further research is needed to elucidate the underlying mechanisms and long-term consequences of precocious puberty under stressful conditions. (Street et al.)

## 5 Circulating levels and bioactivity of miR-30b during pubertal progression in boys

### 5.1 MicroRNAs and pubertal development

MicroRNAs are small non-coding RNA molecules that regulate gene expression. Circulating miRNAs, such as miR-30b, have been implicated in pubertal development. Elevated levels of miR-30b have been associated with testicular function maturation and puberty in boys.

### 5.2 Functional significance

miR-30b regulates genes associated with testicular development and function. Understanding the bioactivity and targets of miR-30b during pubertal progression can provide valuable insights into the molecular mechanisms underlying testicular maturation and hormone regulation (Merup et al.).

## 6 Phthalate exposure and pubertal development: a 15-year follow-up birth cohort study in Taiwan

### 6.1 Environmental endocrine disruptors

Phthalates are ubiquitous environmental chemicals that act as endocrine disruptors, potentially interfering with normal pubertal development. Long-term exposure to phthalates has been associated with alterations in reproductive hormones, the timing of puberty, and adverse health outcomes.

### 6.2 Birth cohort study findings

A 15-year follow-up birth cohort study conducted in Taiwan investigated the association between phthalate exposure and pubertal development. The study revealed potential links between phthalate exposure and altered pubertal timing, hormone levels, and the development of secondary sexual characteristics (Su et al.)

## 7 Timing of puberty and school performance: a population-based study

### 7.1 Academic outcomes and pubertal timing

The timing of puberty has been linked to school performance and academic achievement. Population-based studies have demonstrated that early-maturing individuals may face challenges in educational attainment. In contrast, on-time and late-maturing individuals tend to achieve more favourable academic outcomes.

### 7.2 Implications for educational interventions

Understanding the relationship between pubertal timing and school performance can guide the development of targeted interventions to support adolescents at different stages of pubertal development. Customised strategies can effectively address potential negative impacts and foster positive educational outcomes for adolescents of all backgrounds (Suuatala et al.).

## 8 Conclusion

Puberty encompasses a complex array of neurologic and physiologic changes that significantly impact the development and well-being of individuals. In this editorial, we have explored various facets of puberty, shedding light on the effects of testosterone enanthate and testosterone undecanoate treatment, exploring untargeted metabolomics and lipidomics in central precocious puberty, examining the repercussions of stressful conditions following the COVID-19 epidemic on precocious puberty, investigating the role of miR-30b in boys’ pubertal progression, scrutinising the association between phthalate exposure and pubertal development, and exploring the correlation between the timing of puberty and school performance. By integrating research findings from these diverse domains, we gain a comprehensive understanding of the multifaceted nature of puberty and its profound implications for human development.

## Author contributions

All authors listed have made a substantial, direct, and intellectual contribution to the work and approved it for publication.
